# Development and Evaluation of an In-House ELISA to Detect Anti-Fc*ε*R1*α* IgG Autoantibodies in Chronic Spontaneous Urticaria Patients

**DOI:** 10.1155/2022/6863682

**Published:** 2022-02-25

**Authors:** Chattip Sripatumtong, Tunsuda Tansit, Papapit Tuchinda, Duangjit Kanistanon, Kanokvalai Kulthanan, Yuttana Srinoulprasert

**Affiliations:** ^1^Department of Immunology, Faculty of Medicine Siriraj Hospital, Mahidol University, Bangkok, Thailand; ^2^Department of Dermatology, Faculty of Medicine Siriraj Hospital, Mahidol University, Bangkok, Thailand

## Abstract

**Background:**

Association between chronic spontaneous urticaria (CSU) and autoimmunity has been well documented. Autologous serum skin testing could support the autoimmune etiology of CSU, whereas it is difficult to interpret and could not be performed on antihistamine omitted patients. It was found that immunoglobulin G (IgG) autoantibodies (autoAbs) against high-affinity IgE receptor (Fc*ε*R1) were suggested as a potential trigger in the pathogenesis of CSU. Although many ELISA protocols have been developed to detect these autoAbs, they lacked validation or a reliable cut-off point. We, therefore, aimed to develop a validated ELISA with a reliable cut-off point to quantitate IgG anti-Fc*ε*R1*α* autoAbs for CSU.

**Methods:**

We developed an in-house ELISA to quantitate IgG anti-Fc*ε*R1*α* autoAbs. Sera from 233 CSU patients and 25 healthy people were used to test with ELISA. The cut-off point was obtained from the results subjected to analyze with receiver operating characteristic (ROC) analysis. ELISA was validated with 116 CSU patients and 150 healthy donors.

**Results:**

ELISA revealed that healthy people had a basal level of IgG anti-Fc*ε*R1*α* autoAbs, whereas their levels were significantly lower than autoAbs levels in CSU patients. ROC analysis of ELISA determined the cut-off point at 936.7 ng/ml. Our ELISA was validated and provided excellent sensitivity and specificity at 98.28% and 92.67%, respectively.

**Conclusion:**

Our ELISA could detect significant levels of IgG anti-Fc*ε*R1*α* autoAbs in CSU, supporting that these autoAbs were associated with CSU etiology. Our validated ELISA with the reliable cut-off point provided excellent accuracy at 95.11% (98.28% sensitivity and 92.67% specificity). Our ELISA could be an alternative test benefit for the patient who is unable to omit antihistamine treatment.

## 1. Introduction

Chronic spontaneous urticaria (CSU) is a dermatological condition characterized by recurring wheal and flares lasting more than six weeks with unrecognized etiology. Several hypotheses of CSU etiology have been proposed, and an autoimmune disorder has been suggested as a possible mechanism [1, 2]. Association between CSU and autoreactive antibodies has been revealed and defined as type I and type II hypersensitivity. Type I autoreactive CSU is provoked by immunoglobulin E (IgE) autoantibodies (autoAbs) against self-antigens such as double-strand DNA and thyroid antigens. Type II autoreactive CSU is defined by the effector function of immunoglobulin G (IgG) against IgE or the IgE receptor (FceRI) [3, 4]. These autoAbs were discovered in sera of 23%-46% of CSU patients, and they could induce mediator release from normal donor basophils and skin mast cells [4–7]. Interestingly, IgG autoAbs directed against the Fc*ε*R1*α* chain were also detected in 26%-77% of CSU patients from various cohort studies [6, 8–11]. These reports suggested that autoAbs to the alpha-chain of the high-affinity IgE receptor (Fc*ε*R1*α*) could be a potential trigger in the pathogenesis of CSU.

The methodology used to detect these autoAbs includes in vivo and in vitro tests. An in vivo test by intradermal injection of a patient's serum into themselves, so-called autologous serum skin testing (ASST), could theoretically support the autoimmune etiology of CSU [7, 12]. ASST was positive in 25%-65% of CSU patients [6, 12, 13]. However, it was difficult to interpret because it provided little or no information about functional components. A significant proportion of patients with allergic or nonallergic rhinitis, multiple drug allergy syndromes, and even healthy control subjects were positive with ASST [14]. Unluckily, ASST could not be done on CSU patients who could not discontinue antihistamine therapy. In vitro techniques such as histamine release (HR) from basophils and western blotting/immunoprecipitation were employed as an alternative method to identify autoAbs [6, 8]. These laborious procedures are unsuitable for screening purposes and have limited utility for determining autoAbs titers. Many groups of researchers ever developed in-house ELISA, and these results revealed the presence of IgG anti-Fc*ε*R1*α* autoAbs [15–17]. However, how to earn and apply cut-off points was never clearly demonstrated. We, therefore, aimed to develop a validated ELISA with a reliable cut-off point to quantitate IgG anti-Fc*ε*R1*α* autoAbs for CSU serum samples.

## 2. Materials and Methods

### 2.1. Experimental Design

Sera from 233 CSU patients who attended the Urticaria Clinic (Siriraj Urticaria Center of Reference and Excellence; UCARE), Department of Dermatology, Siriraj Hospital, Bangkok, Thailand, were collected from August 2008 to June 2019. All patients underwent autologous serum skin testing (ASST), and the patients were divided according to the results into the ASST positive and ASST negative groups. Twenty-five healthy individuals without CSU and allergic/autoimmune diseases were also recruited as a control group. These sera from CSU and healthy people were used to find out the cut-off point of the ELISA test. The second cohort of sera was collected from 116 CSU patients who attended the same Urticaria Clinic from August 2018 to June 2021. One hundred and fifty healthy donors were recruited from September 2019 to January 2020. The second cohort group was used to validate the ELISA test. All of the participants in this study provided written informed consent.

### 2.2. Patient Sample Collection and Preparation for ELISA

Clot blood samples were obtained in sterile conditions from all volunteers. The clot blood samples were subjected to centrifuge at 3000 rpm for 15 minutes at room temperature to obtain sera. The sera were aliquoted and kept at -20°C until use.

### 2.3. ELISA for Detecting of Human Fc*ε*R1*α* autoAbs

In-house enzyme-linked immunosorbent assay (ELISA) was developed for detecting anti-Fc*ε*R1*α* auto-antibody. A 96-well ELISA plate (Costar, Kennebunk, MC) was coated with 0.00035 mg/ml of human Fc*ε*R1alpha protein (Sino Biological, Wayne, PA). After the plate was kept overnight at 4°C, it was washed five times with PBS-Tween and blocked with 3% skim milk for 1 hour at 37°C. After washing again, a 50 *μ*l serum sample diluted with 1% skim milk buffer to final dilution at 1 : 100 dilution was added into each well; then, the plate was incubated at 37°C for one hour. After incubation, the plate was washed; meanwhile, HRP-conjugated rabbit anti-hIgG (Dako, Santa Clara, CA) was freshly prepared final dilution at 1 : 1,000 and added to all sample wells. The plate was incubated at 37°C for one hour and washed after all. To measure the amount of anti-Fc*ε*R1*α* auto-antibody, the reaction was developed to detect color using 3,3′,5,5′-tetramethylbenzidine (Abcam, Cambridge, UK) in substrate solution at room temperature in a dark chamber for 10 min. The reaction was stopped with 1 N HCl, and optical density (OD) was read at 450 nm with a microplate spectrophotometer (Biotek, Winooski, VT). To quantify human IgG anti-Fc*ε*R1*α* autoAbs, human immunoglobulin G (hIgG) range from 0.25 to 0.0019 *μ*g/ml was also used to establish a standard curve.

### 2.4. Statistical Calculations

Both parametric (*t*-test or paired *t*-test) and nonparametric (sign test or signed-rank test) were used as appropriate. A *p* value of less than 0.05 was considered to be statistically significant. The amount of hIgG anti-Fc*ε*R1*α* in every sample was calculated from the hIgG standard curve of each corresponding performed experiment. The cut-off value was obtained by applying receiver operating characteristic (ROC) data analysis of hIgG in sera of CSU and healthy donors. Validation of Fc*ε*R1*α* ELISA was performed with 14 CSU-ASST positive, 102 CSU-ASST negative, and 150 healthy control samples. Values for sensitivity, specificity, positive and negative predictability (PPV and NPV, respectively), and accuracy were calculated.

### 2.5. Ethical Approvals

This study was approved by the Ethical Committee on Research Involving Human Subjects, Faculty of Medicine, Siriraj Hospital, Mahidol University (COA No. Si 540/2016).

## 3. Results

### 3.1. Patient Demographics

We recruited two hundred and fifty-eight volunteers, as shown in Table 1. The healthy group contained eighteen females and seven males with a mean age of 39.5 years, the ASST positive group had eighty females and eighteen males with a mean age of 35.7 years, and the ASST negative group included a hundred females and thirty-five males with a mean age of 38.3 years. The mean age of the three groups was statistically insignificant.

### 3.2. ELISA-Based Detection of Human IgG Anti-Fc*ε*R1*α* autoAbs

**Figure 1:**
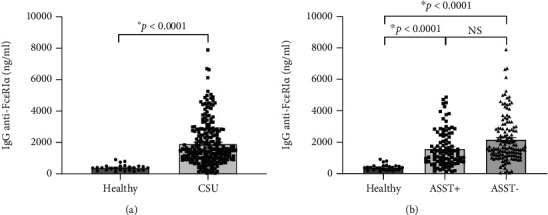
Overlay of levels of hIgG anti-Fc*ε*RI*α* in sera from healthy and CSU donors. (a, b) Bar graphs overlayed with scatter plots represent levels of human IgG against anti-Fc*ε*R1*α* in healthy groups, ASST positive, and ASST negative (mean ± SEM). ^∗^A *p* value of less than 0.05 was statistically significant.

Basal levels of human IgG (hIgG) against the whole molecule of Fc*ε*R1*α* were detected in sera of the healthy group, as shown in Figure 1(a). Sera from CSU contained a higher level of hIgG anti-Fc*ε*R1*α* autoAbs (1,928.19 ± 91.48; mean ± SEM) as compared with sera from healthy donors (400.21 ± 38.90; mean ± SEM) (Figure 1(a)), as well as the levels of hIgG anti-Fc*ε*R1*α* autoAbs in sera from both ASST positive (1,589.64 ± 118.21; mean ± SEM) and ASST negative (2173.95 ± 128.82; mean ± SEM) CSU patients were also significantly higher than that of healthy subjects (Figure 1(b)). However, the levels of hIgG anti-Fc*ε*R1*α* autoAbs among CSU cohorts (ASST positive and ASST negative) were not significantly different even the levels of IgG anti-Fc*ε*R1*α* autoAbs in sera from ASST negative CSU trend to higher than that of ASST positive CSU as shown in Figure 1(b).

### 3.3. Validation of IgG Anti-Fc*ε*R1*α* ELISA

To obtain cut-off point of hIgG anti-Fc*ε*R1*α* for discrimination between healthy people and CSU patients, levels of hIgG anti-Fc*ε*R1*α* were subjected to analyze by receiver operating characteristic (ROC) analysis. ROC analysis yielded sensitivity and sensitivity of each level of hIgG anti-Fc*ε*R1*α* in two populations for further validation. Youden's index derived from sensitivity and sensitivity of each level of hIgG anti-Fc*ε*R1*α* was demonstrated in Table 2 used to suggest optimal cut-off point. The highest value of Youden's index at 167.30 was chosen to achieve an optimal cut-off value of hIgG anti-Fc*ε*R1*α* at 936.7 ng/ml.

**Figure 2:**
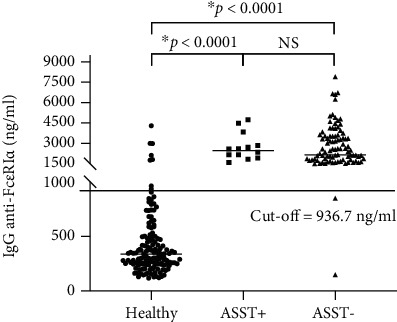
Levels of hIgG anti-Fc*ε*RI*α* in sera from the second cohort of healthy and CSU volunteers. Scatter plot represents levels of human IgG against anti-Fc*ε*R1*α* in healthy (*n* = 150), ASST positive (*n* = 14), and ASST negative (*n* = 102) (mean ± SEM). ^∗^A *p* value of less than 0.05 was statistically significant. The cut-off line was set at 936.7 ng/ml.

To validate the cut-off point at 936.7 ng/ml, additional 266 serum samples were from healthy people (*n* = 150); CSU patients (ASST positive, *n* = 14, and ASST negative, *n* = 102) were used to test. Sensitivity, specificity, positive predictive value, and negative predictive value were calculated and revealed at 98.28%, 92.67%, 91.20%, and 98.58%, respectively. The accuracy of the in-house ELISA was at 95.11%, as shown in Figure 2 and Table 3.

## 4. Discussion

The autoimmune etiology of CSU was corroborated by an in vivo investigation, so-called the autologous serum skin test (ASST), which is an intradermal injection of a patient's serum into their own skin [12]. Wheal and flare reactions indicate a positive result of ASST. Our study found that frequency of ASST positivity in CSU patients was 42% and 12% in the first and second cohort studies, respectively. It was consistent with the previous studies that the positive rate of ASST results in CU patients was various (34%-70%) [12, 18–22]. Female predominance among CSU cases was also observed in our study and other previous reports [18, 20, 23]. Nevertheless, positive ASST can be observed in clinical remission of CSU patients, allergic and nonallergic patients, autoimmune patients, and even healthy people [14, 24]. Apart from the difficulty of ASST interpretation, another problematic obstruction to performing ASST is antihistamine omission in some patients. False-positive ASST can occur due to improper preparation of serum samples [24, 25]. Additionally, ASST cannot indicate which factors in serum cause mast cell degranulation. Therefore, identifying factors causing mast cell degranulation in a patient's serum using in vitro test could be an alternative investigation to solve the above issues.

Factors, which circulate in CSU blood and cause mast cell degranulation by induction of wheal and flare reactions in response to ASST, have been demonstrated [26]. These factors can be both IgE dependence and non-IgE dependence. One non-IgE dependent factor was determined as IgG autoAbs against the alpha subunit of the high-affinity Fc*ε*R1 in sera of CSU patients [6]. It was revealed that human IgG anti-Fc*ε*R1*α* could induce histamine release irrespective of IgE sensitization of the basophils, causing a pathological effect. These autoAbs against Fc*ε*R1*α* can provoke chronic stimulation and degranulation of these cells in an IgE-independent fashion [27]. As proof of concept, Hide and colleagues demonstrated that preincubating donor basophils with the soluble fragment of Fc*ε*R1*α* before the addition of purified IgG from sera of patients with CSU was able to neutralize histamine release from basophils. The concept that circulating IgG antibodies against high-affinity IgE receptor Fc*ε*R1*α* likely contribute to the pathogenesis of CSU has become widely accepted [4]. Human IgG autoAbs against Fc*ε*R1*α* can be detected in 24%-64% of sera from CSU patients [10, 15, 23, 28]. Interestingly, our study demonstrated that the higher prevalence of IgG against Fc*ε*R1*α* was 76.4% and 98.3% in the first and second CSU cohorts. Meanwhile, our study also showed 7% of normal control (NC) sera contained levels of IgG anti-Fc*ε*R1*α* above the cut-off point. The presence of IgG against Fc*ε*R1*α* in NC sera was consistent with previous reports [23, 29]. A discrepancy of findings could be due to various methods, and the cut-off point in previous studies has never been stated and quantified. Our in-house ELISA was developed and performed with a large cohort to obtain a statistical cut-off point. The newly recruited cohort validated the test with a quantified cut-off point according to the epidemiologic process. Even the presence of Fc*ε*R1*α* autoAbs was noted in the sera of patients with other autoimmune skin conditions and even in healthy subjects, though they did not manifest wheal and flare reactions [15]. However, the levels of Fc*ε*R1*α* autoAbs were below the cut-off in healthy and other autoimmune disorders, as shown in Figures 1 and 2, and Supplement Figure (available here). Our in-house ELISA test could differentiate levels of anti-Fc*ε*R1*α* antibodies in CSU patients from those levels in other autoimmune populations and normal healthy people, which it can be used to screen sera from large patient cohorts. As results from ELISA are informative data, which can be used for further epidemiologic study. The limitation of this research is that ELISA is not a functional test.

Many reports demonstrated an association between the presence of IgG against Fc*ε*R1*α* and ASST positivity [30–32]. Recently, Ulambayar and colleagues reported that positivity rates for IgG autoAbs to Fc*ε*R1*α* were higher in ASST positive CSU patients than in ASST negative CSU patients [23]. Anti-Fc*ε*R1*α* autoAbs were detected in approximately 40% of CSU sera as well as its higher frequency of positivity in CSU patients was demonstrated in ASST positive sera [23, 33]. As shown in Figure 2 and Table 3, we found that significant levels of IgG against Fc*ε*R1*α* were detected in all ASST positive sera, which this finding strongly supported those reports. Interestingly, our study revealed that levels of anti-Fc*ε*R1*α* autoAbs were not statistically significant between ASST positive and ASST negative groups, as shown in Figures 1(b) and 2. Nevertheless, our finding was consistent with another report demonstrated that levels of autoAbs in CSU did not correlate with disease activity [29].

In conclusion, our ELISA detected significant levels of human IgG against Fc*ε*R1*α* in CSU sera, indicating that IgG anti-Fc*ε*R1*α* autoAbs are associated with CSU. Our finding suggested that IgG anti-Fc*ε*R1*α* could be a potential CSU biomarker and may predict outcomes of the new biologic treatment in the further study [34]. Importantly, our test might be an appropriate in vitro test, overcoming a limitation of ASST benefit for patients who cannot avoid antihistamine treatment.

## Figures and Tables

**Table 1:** Demographic data of volunteers.

Groups	Healthy	CSU	
		ASST+	ASST-
Patient no.	25	98	135
Age $\left(\bar{x}\pm SD\right)$	28.4 ± 7.5	35.7 ± 12.6	38.3 ± 12.7
Gender M/F (%)	28.0/72.0	18.4/81.6	25.9/74.1

**Table 2:** Youden's index determines cut-off values.

Cut off value	Sensitivity	Specificity	Youden's index
>920.0	77.25	87.27	164.52
>922.2	77.25	89.09	166.34
>927.0	76.82	89.09	165.91
>931.2	76.39	89.09	165.48
>936.7	76.39	90.91	167.30
>942.1	75.97	90.91	166.88
>949.7	75.11	90.91	166.02
>963.8	74.68	90.91	165.59
>978.0	74.25	90.91	165.16

**Table 3:** Validation of cut-off value in the second cohort.

	CSU	Healthy	Total	
ELISA +	114	11	125	PPV 91.20%
ELISA -	2	139	141	NPV 98.58%
Total	116	150	266	
	Sensitivity 98.28%	Specificity 92.67%		

^∗^Cut-off value was at 936.7 ng/ml.

## Data Availability

The datasets generated and analyzed for the present study are available from the corresponding author on reasonable request.

## Supplementary Materials

Supplementary Materials Levels of hIgG anti-Fc*ε*R1*α* autoantibodies in sera from healthy, CSU, and autoimmune patients. Scatter plot represents levels of human IgG against anti-Fc*ε*R1*α* in healthy (25; 400.2 ± 38.90 ng/ml), CSU patients (116; 2,710 ± 128.6 ng/ml), and autoimmune patients (30; 914.7 ± 127.3 ng/ml) (*n*; mean ± SEM ng/ml). The cut-off line was set at 936.7 ng/ml. Levels of hIgG anti-Fc*ε*R1*α* of most CSU were over cut-off point and significantly higher than that of autoimmune and healthy people. Levels of hIgG anti-Fc*ε*R1*α* of autoimmune patients were significantly higher than that of healthy people.
