# Supplementary figures and images for: Development and Evaluation of an In-House ELISA to Detect Anti-FcεR1α IgG Autoantibodies in Chronic Spontaneous Urticaria Patients
Source: J Immunol Res. 2022 Feb 25;2022:6863682. doi: 10.1155/2022/6863682 (PMC8896930; doi:10.1155/2022/6863682)

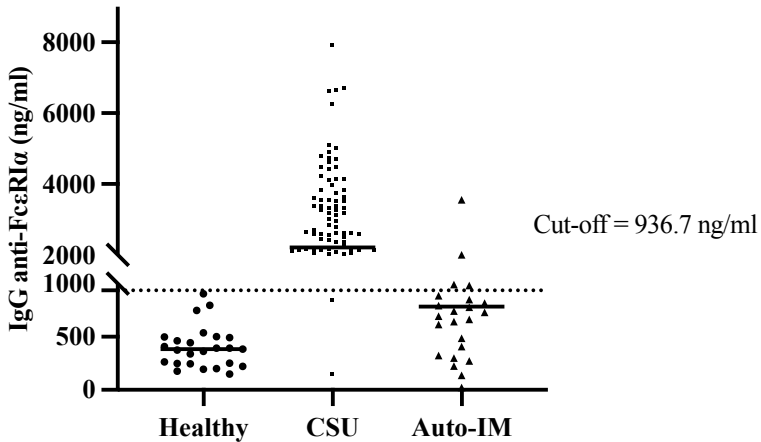

Supplement: Supplementary Materials — Levels of hIgG anti-FcεR1α autoantibodies in sera from healthy, CSU, and autoimmune patients. Scatter plot represents levels of human IgG against anti-FcεR1α in healthy (25; 400.2 ± 38.90 ng/ml), CSU patients (116; 2,710 ± 128.6 ng/ml), and autoimmune patients (30; 914.7 ± 127.3 ng/ml) (n; mean ± SEM ng/ml). The cut-off line was set at 936.7 ng/ml. Levels of hIgG anti-FcεR1α of most CSU were over cut-off point and significantly higher than that of autoimmune and healthy people. Levels of hIgG anti-FcεR1α of autoimmune patients were significantly higher than that of healthy people. [file 6863682.f1.pdf]
